# Subsyndromal Delirium in Critically Ill Patients—Cognitive and Functional Long-Term Outcomes

**DOI:** 10.3390/jcm12196363

**Published:** 2023-10-04

**Authors:** Maria Carolina Paulino, Catarina Conceição, Joana Silvestre, Maria Inês Lopes, Hernâni Gonçalves, Cláudia Camila Dias, Rodrigo Serafim, Jorge I. F. Salluh, Pedro Póvoa

**Affiliations:** 1 NOVA Medical School, New University of Lisbon, 1150-082 Lisbon, Portugal; joanapsilvestre@gmail.com (J.S.); pedrorpovoa@gmail.com (P.P.); 2 Department of Intensive Care, Hospital de São Francisco Xavier, Centro Hospitalar de Lisboa Ocidental, 1150-199 Lisbon, Portugal; catarinapconceicao@gmail.com; 3 Department of Intensive Care, Hospital da Luz Lisboa, 1500-650 Lisbon, Portugal; mariaines.nolopes@gmail.com; 4 Lisbon School of Medicine, University of Lisbon (FMUL), 1649-028 Lisbon, Portugal; 5 Department of Intensive Care, Hospital dos Lusíadas, 1500-458 Lisbon, Portugal; 6 Center for Health Technology and Services Research (CINTESIS@RISE), Faculty of Medicine, University of Porto, 4200-450 Porto, Portugal; hernanigoncalves@med.up.pt (H.G.); camila@med.up.pt (C.C.D.); 7 Department of Community Medicine, Information and Health Decision Sciences (MEDCIDS), Faculty of Medicine, University of Porto, 4200-450 Porto, Portugal; 8 D’OR Institute for Research and Education, Rio de Janeiro 22281-100, Brazil; rodrigobserafim@gmail.com (R.S.); jorgesalluh@gmail.com (J.I.F.S.); 9 Post-Graduate Program, Federal University of Rio de Janeiro, Rio de Janeiro 21941-913, Brazil; 10 Center for Clinical Epidemiology and Research Unit of Clinical Epidemiology, OUH Odense University Hospital, C 5000 Odense, Denmark

**Keywords:** subsyndromal delirium, delirium, cognitive dysfunction, ICU, long-term outcome

## Abstract

Subsyndromal delirium (SSD) in the Intensive Care Unit (ICU) is associated with an increased morbidity with unknown post-discharge functional and cognitive outcomes. We performed a prospective multicenter study to analyze the mental status of patients during their first 72 h after ICU admission and its trajectory, with follow-ups at 3 and 6 months after hospital discharge. Amongst the 106 included patients, SSD occurred in 24.5% (n = 26) and was associated with the duration of mechanical ventilation (*p* = 0.003) and the length of the ICU stay (*p* = 0.002). After the initial 72 h, most of the SSD patients (30.8%) improved and no longer had SSD; 19.2% continued to experience SSD and one patient (3.8%) progressed to delirium. The post-hospital discharge survival rate for the SSD patients was 100% at 3 months and 87.5% at 6 months. At admission, 96.2% of the SSD patients were fully independent in daily living activities, 66.7% at 3-month follow-up, and 100% at 6-month follow-up. Most SSD patients demonstrated a cognitive decline from admission to 3-month follow-up and improved at 6 months (IQCODE-SF: admission 3.13, *p* < 0.001; 3 months 3.41, *p* = 0.019; 6 months 3.19, *p* = 0.194). We concluded that early SSD is associated with worse outcomes, mainly a transitory cognitive decline after hospital discharge at 3 months, with an improvement at 6 months. This highlights the need to prevent and identify this condition during ICU stays.

## 1. Introduction

Subsyndromal delirium (SSD) is commonly considered as an intermediate stage of severity between delirium and non-delirium [1,2]. There is no universal consensus on the definition and diagnosis criteria for SSD. In the fifth edition of the Diagnostic and Statistical Manual of Mental Disorders (DSM-5) this condition was referred to as an “attenuated delirium syndrome”, without specific criteria and terminology [3]. In the updated DSM-5-TR the term “Subsyndromal Delirium” was introduced as “other specified delirium” [4]. To date, there is no dedicated assessment tool for SSD in Intensive Care Units (ICU). Some proposals have suggested using the same assessment scales that are used for delirium assessments. The Intensive Care Delirium Screening Checklist (ICDSC), which is a quantitative tool developed to diagnose and grade delirium symptoms, has incorporated a scoring component indicative of SSD (typically between 1 and 3 points), providing a structured means for assessing and monitoring this condition.

The management of delirium and SSD in ICUs remains a challenge. The authors of the Guidelines for the Prevention and Management of Pain, Agitation, Sedation, Delirium, Immobility, and Sleep Disruption (PADIS) recommend a multicomponent, nonpharmacological approach to reducing the risk factors for delirium, enhancing cognitive function, and improving sleep, mobility, and patient reorientation [5]. Innovative concepts and ICU design approaches aimed at stress reduction and supporting the healing process of patients are also being explored [6]. A pharmacologic strategy for preventing and treating delirium has not definitively been recommended. The literature on this topic yields conflicting results, and study comparations are difficult because of different methodology assessments. Studies comparing antipsychotics to placebos have shown some benefit for quetiapine compared to ziprasidone or haloperidol, and combination therapies of dexmedetomidine plus haloperidol and quetiapine with haloperidol have demonstrated potential benefits [7]. Melatonin and ramelteon do not seem to reduce the delirium incidence in ICU patients [8]. To date, studies attempting to pharmacologically prevent the transition from SSD to delirium have yielded conflicting findings. Al-Qadheeb et al. reported that low-dose haloperidol administered to mechanically ventilated critically ill adults did not prevent the progression of SSD to delirium, but did reduce the duration of agitation in the haloperidol group [9]. In contrast, Hakim et al. observed that the administration of risperidone to elderly patients who had undergone on-pump cardiac surgery and were experiencing SSD was associated with a significantly lower incidence of delirium compared to that in a placebo group (13.7% vs. 34%, *p* = 0.031) [10].

Delirium studies on critically ill adults have consistently found an association with cognitive impairment at 3 and 12 months after ICU discharge, but its association with depression, functionality/dependence, or mortality has not been consistently established [11,12,13].

The functional and cognitive outcomes of SSD are not as well understood as those of delirium. The existing literature on SSD has primarily focused on non-critically ill patients, where it has a combined prevalence of 23% (95% CI, 9–42%) [14] and outcomes that fall between those with delirium and those with no symptoms of delirium [15]. In this population, SSD is associated with longer hospital stays, poor functional and cognitive outcomes after discharge, a higher mortality, and an increased risk of institutionalization [12,13,14,15,16]. Postoperative subsyndromal delirium has also been linked to a worsened functional status one month after surgery [16].

In the ICU, studies on SSD outcomes are limited. SSD is associated with longer ICU stays, lower cognitive scores, and an increased likelihood of institutionalization [17,18]. It does not seem to be associated with ICU or hospital mortality and the duration of mechanical ventilation [17]. One study documented that once delirium or SSD occurred after elective coronary artery bypass graft surgery, it was associated with a significant short-term decline in cognitive performance at ICU discharge [19]. However, there is a lack of information regarding long-term follow-ups of SSD in critically ill patients [18,20].

The present study aims to evaluate the prevalence of early SSD in critically ill patients and cognitive and functional follow-ups at 3 and 6 months after hospital discharge.

## 2. Materials and Methods

We conducted a prospective, observational, multicenter clinical study (SubSynd study) in four medical and surgical Portuguese ICUs, between 1 August 2018 and 31 March 2020, with the ClinicalTrials.gov Identifier: NCT0381345. 

All adult patients admitted to the ICU were screened for the exclusion criteria. The exclusion criteria included the following: acute neurologic injury as the primary reason for ICU admission (neurocritical patient with a Glasgow Coma Scale (GCS) of <14 at admission or in the days prior); a history of severe cognitive dysfunction or severe dementia; impaired hearing or visual acuity; speech difficulties or an inability to speak or understand Portuguese; patients expected to die within 24 h after admission; a limitation of therapeutic effort; refusal to participate; an inability to provide informed consent; and patients who were readmitted to the ICU. Patients with an RASS score below three and patients with missing evaluations (ICDSC assessment) within the first 72 h after admission were also excluded.

We used the ICDSC scale for the diagnosis of SDD and applied it until ICU discharge or death, for a maximum duration of 14 days. Patients who obtained a score of 0 on each examination were categorized as non-delirious, those scoring between 1 and 3 were classified as having SSD, and a score of 4 or higher was indicative of delirium. The ICDSC, in conjunction with the Richmond Agitation and Sedation Scale (RASS), was applied once daily, with an initial assessment by a clinician occurring in the morning. If the patient was deeply sedated (RASS < −3), the assessment could not be conducted. All the clinicians involved in the screening for SSD and delirium underwent systematic training by a senior instructor to ensure the proper utilization of the ICDSC. 

For the specific analysis conducted in this study, our focus was on the early occurrence of SSD. We assessed the patients within the first 72 h following their ICU admission and analyzed the progression of each patient group over the subsequent three days (4th, 5th, and 6th days after admission). The patients were categorized in the first 72 h, based on the highest score of the ICDSC, as having (1) delirium; (2) SSD; or (3) no-delirium/no-SSD. This means that patients with at least one classification of delirium in the first 72 h were classified as having delirium. Patients without delirium, but with at least one classification of subsyndromal delirium, were classified as having SSD. Patients without delirium or subsyndromal delirium were classified as no-delirium/no-SSD. We selected this period (72 h) since SSD and delirium occurrence is higher in the first days after ICU admission [21,22]. 

### 2.1. Outcome and Endpoints

At enrollment, we collected the following baseline demographic variables: age, gender, pre-illness cognitive status (via IQCODE), pre-illness disability status (via the Katz index), medical comorbidities (Charlson comorbidity index), admission type (urgent or elective), admission characterization, pre-ICU location, admission diagnosis, E-PRE-DELIRIC Score [23], APACHE II score, and SOFA score. We also recorded the use of sedative and analgesic intravenous medication, corticosteroids, renal replacement therapy, and C-reactive protein levels. 

The primary outcome of the study was the prevalence of early-onset SSD (within the first 72 h). The secondary outcomes included the duration of mechanical ventilation, the length of the stay in the ICU and hospital, ICU and hospital mortality rates, and cognitive and functional outcomes at 3 and 6 months after hospital discharge.

### 2.2. Functional and Cognitive Follow-Up

We assessed the functional and cognitive outcomes at 3 and 6 months post-hospital discharge. The functional evaluation was conducted using the modified Katz index of independence in activities of daily living [24,25], while the cognitive evaluation utilized the short form of the Informant Questionnaire on Cognitive Decline in the Elderly (IQCODE-SF) [26]. These assessments were performed via phone calls, either to the patients themselves or their proxies. To maintain consistency, all the phone calls were conducted by the principal investigator.

The IQCODE was originally described as a 26-item informant questionnaire that retrospectively assesses changes in cognitive performance over a 10-year time period and allows a screen for potential dementia. Later, a shorter version was created, the IQCODE-Short form (IQCODE-SF), measuring a range of cognitive declines using a 16-item score. This short form is common in clinical practice and has been recommended as the preferred IQCODE format, being repeatedly shown to be effective in identifying the presence of significant cognitive impairments in medical populations and elderly populations [27]. For each scale item, the scores change on a five-point ordinal hierarchical scale, with scores ranging from 1.0 (much improved) to 5.0 (much worse) and 3.0 indicating no change. The total score for the 16 questions is then divided by 16 to generate a score ranging from 1 to 5, with higher scores denoting worsening cognitive function. 

In the original IQCODE development and validation work, an average score higher than 3.31 was indicative of cognitive impairment [27], but there is no consensus on the optimal threshold and no guidance for the use of subthreshold IQCODE scores [28]. With the lack of a consensual threshold, we decided to use the same cut-off already used in a mixed population of ICU patients reported by P.P. Pandharipande et al., where patients were divided into five groups: scores of <3 indicating improvement, a score = 3.0 indicating no change, scores of >3 but <3.3 indicating cognitive decline, scores of ≥3.3 indicating an increased likelihood of cognitive impairment, and scores of ≥3.6 indicating preexisting cognitive impairment [29]. The IQCODE-SF can be used not only to predict potential dementia, but also as a first assessment of cognitive function and screening for mild cognitive impairment [30]. The limited available data suggest that it is sensitive, but not sufficiently specific to inform clinical decision making. In this situation, the additional complementation of the IQCODE is needed, with a more specific assessment.

The Katz Index of Independence in Activities of Daily Living (Katz ADL Index) evaluates the adequacy of performance in six functions: bathing, dressing, toileting, transferring, continence, and feeding. Each function is scored as either independent (yes) or dependent (no). Based on their total score, the patients were categorized as follows: total dependency = 0; severe dependence = 1–2; moderate dependence = 3–4; mild dependence = 5; and total independence = 6.

### 2.3. Ethics

The study received ethical approval from the Research and Ethics Committee of Centro Hospitalar de Lisboa Ocidental (nº 20170700050, date of approval 7 June 2017) and from the National Data Protection Commission (nº 4685/2017, date of approval 28 April 2017).

Each participating hospital obtained approval from their respective Institutional Review Boards. Written informed consent was obtained from the participants or their proxies and no incentives were provided to encourage participation. The study was conducted in accordance with the approved ethical guidelines. There were no interventions in the regular management of the patients. Data were collected and entered into a dedicated electronic database that was specifically created for this study. The authors ensured the confidentiality of the data, and the patients were assigned unique code numbers to maintain their anonymity. 

### 2.4. Data Analysis and Statistical Methods

Standard descriptive statistics were used as appropriate. Continuous variables were expressed as mean ± standard deviation or median and interquartile range, depending on the normality assumption. Categorial variables were expressed as absolute (n) and relative (%) frequencies.

The chi-square test was used to evaluate the associations between the categorical variables, while, for continuous variables and group comparisons (patients with SSD, delirium, and no-delirium/no-SSD), the t-Student and Kruskal–Wallis tests were used. The variables were compared between the three groups using the Bonferroni test. We used a multivariable regression to evaluate the association between early SSD, invasive mechanical ventilation, and the length of the ICU stay. Covaried APACHE II, E-PRE-DELIRIC score, age, SOFA score, and admission characterization were used (variables with a *p* < 0.25 in a univariable analysis). The significance level used was 0.05. All the statistical analyses were performed with the IBM SPSS Statistics software, version 26.0 (IBM, Somers, NY, USA).

## 3. Results

**Figure 1:**
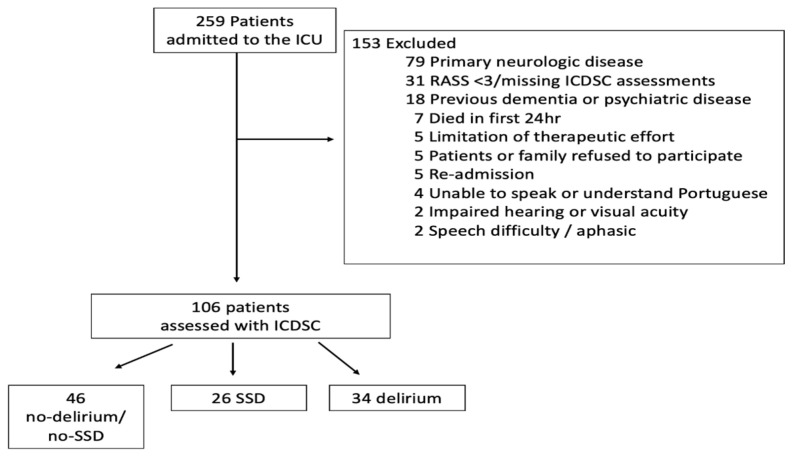
Flow of patients in study cohort. ICU—Intensive Care Unit; RASS—Richmond Agitation-Sedation Scale; ICDSC—Intensive Care Delirium Screening Checklist; and SSD—Subsyndromal delirium.

Out of the initial 259 patients screened, 106 patients underwent daily assessments. Using their ICDSC score within the first 72 h, these patients were categorized into three groups: 26 patients with SSD (24.5%), 34 patients with delirium (32.1%), and 43 patients without delirium or SSD (34%) (Figure 1).

Their demographic characteristics are presented in Table 1. There was a predominance of male patients and a significative increase in age in the groups from no-delirium/no-SSD to SSD and delirium (61 years, 67 years, and 74 years, respectively; *p* = 0.003). The type of admission was mainly urgent (*p* = 0.17), with a predominance of medical admissions (*p* = 0.09) with infections/sepsis being the most prevalent diagnosis. The severity of the illness expressed by the median APACHE II score and median admission SOFA score showed an increase through the three groups (no-delirium/no-SSD < SSD < delirium), higher in delirium patients, with a significative association with both scores (*p* < 0.001) (Table 1). The median (IQR) for the E-PRE-DELIRIC score was significantly higher for delirium 0.83 (0.66–0.89) when compared to SSD 0.58 (0.47–0.68) and no-delirium/no-SSD 0.56 (0.33–0.73), presenting a significative association not only with delirium, but also with SSD occurrence (*p* < 0.001). 

The median (IQR) of the number of days of mechanical ventilation was significantly associated with cognitive dysfunction (no-delirium/no-SSD group 0 (0–1)); SSD, 0.5 (0–1); and delirium, 1.5 (0–7; *p* = 0.003). The median length of ICU stay was longer in the delirium group compared to that in the SSD group and no-delirium/no-SSD group (8 (4–10) days, 4.5 (3–6) days, and 4 (2–6) days, respectively; *p* = 0.002). The length of hospital stay was similar in the three groups (17 days, 20.5 days, and 23 days, respectively; *p* = 0.22). No significant differences were observed among the three groups concerning either hospital or ICU mortality (*p* = 0.41 and *p* = 0.68) (Table 2). 

**Figure 2:**
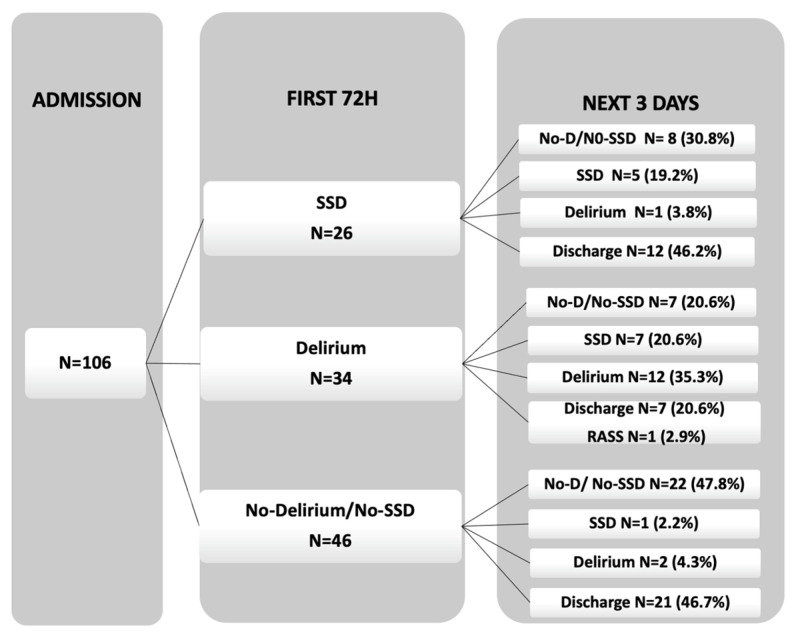
Cognitive trajectory in the three days after the first 72 h of admission.

The cognitive trajectory during days 4, 5, and 6 after admission is presented in Figure 2. Among the no-delirium/no-SSD patients, a significant number remained without delirium and without SSD (22 patients, 47.8%) or were discharged to the ward (21 patients, 46.7%). In the SSD group, most patients showed a rapid improvement to no-delirium/no-SSD (8 patients, 30.8%) or were discharged (12 patients, 46.2%), while 5 patients (19.2%) continued to have SSD, and only 1 patient (3.8%) progressed to delirium. The rapid resolution of this early SSD is another fact that points to its greater proximity to patients with normal cognition than to delirium patients. Among the delirium group, most patients remained with delirium (12 patients, 35.3%), while others either developed SSD (7 patients, 20.6%), improved to no-delirium/no-SSD (7 patients, 20.6%), or were discharged (7 patients, 20.6%). Only one patient was not assessed for having an RASS of <3. 

### 3.1. Follow-Ups 3 and 6 Months after Hospital Discharge

At 3 months post-discharge, all the SSD patients were still alive and there were no statistically significant differences in the mortality rates among the three groups (no-delirium/no-SSD: 2.7%; SSD: 0%; and delirium: 10%; *p* = 0.43). Concerning readmissions, only two patients (13.3%) from the SSD group required hospital re-admission, and there were also no statistically significant differences among the groups (no-delirium/no-SSD: 22.2%; SSD: 13.3%; and delirium: 29.4%; *p* = 0.63) (Table 3, Figure S1).

Regarding the risk of institutionalization, most SSD patients were able to return home (14 patients, 93.3%), while only 1 patient (6.7%) went to a nursing home. Similarly, in the other groups, most patients were able to return home (no-delirium/no-SSD: 88.9%; and delirium: 70.6%). Only three delirium patients were still in the hospital at the time of the evaluation. At 6 months post-discharge, the mortality rate among the SSD patients was 12.5% (two patients), among no-delirium/no-SSD was 5.9% (two patients), and no deaths occurred among the delirium patients (*p* = 0.35). The rate of hospital re-admission at 6 months was higher in the delirium group (six patients, 40%). In terms of the risk of institutionalization, most patients were still able to reside at home (no-delirium/no-SSD: 31 patients, 96.9%; SSD: 13 patients, 92.9%; and delirium: 14 patients, 93.3%; *p* > 0.99) (Table 3).

### 3.2. Functional Outcomes 3 and 6 Months Post-Discharge

At ICU admission, most patients in all three groups were fully independent (no-delirium/no-SSD: 36 patients, 78.3%; SSD patients: 25 patients, 96.2%; and delirium: 21 patients, 61.8%). The patients that were more dependent at hospital admission were those who developed delirium (Table 4).

At 3 and 6 months after discharge, the SSD patients exhibited the best functional outcomes, followed by the no-SSD/no-delirium patients. At 3-month follow-up, 10 SSD patients (66.7%) were fully independent, 2 patients (13.3%) had severe dependence, and 3 patients (20%) were completely dependent. However, upon reevaluation at 6 months, all the SSD patients had regained their full independence.

### 3.3. Cognitive Outcomes 3 and 6 Months after Hospital Discharge

**Figure 3:**
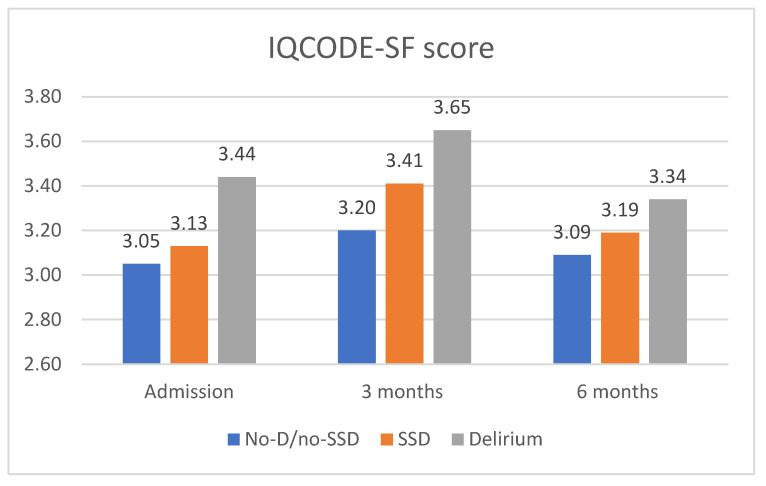
Short Informant Questionnaire on Cognitive Decline in the Elderly (IQCODE-SF score), 3 and 6 months after discharge, mean values. Score < 3 indicating improvement; score = 3.0 no change; score > 3 but <3.3 indicating decline in cognition; score ≥ 3.3 indicates an increased probability of cognitive impairment; and score ≥ 3.6 indicates preexisting cognitive impairment. *p*-value at admission <0.001; *p*-value at 3 months 0.02; and *p*-value 6 months 0.19 (Kruskall–Wallis test). Legend: No-D/no-SSD—No-delirium/No-Subsyndromal delirium; and SSD—Subsyndromal delirium.

Regarding cognitive outcomes, the SSD patients exhibited a decline in their cognitive function from admission to the 3-month follow-up (Figure 3). At admission, the IQCODE-SF score was 3.13, already indicating cognitive decline. This score increased to 3.41 at 3 months, indicating cognitive impairment. At the 6-month follow-up, the IQCODE-SF score was 3.19, slightly lower than that at 3 months. When compared to the other groups, the SSD patients had IQCODE-SF scores in an intermediate range between the other two groups, but closer to the no-delirium/no-SSD group.

The no-delirium/no-SSD patients did not show significant changes in their cognitive function at admission, but experienced worsening at the 3-month follow-up, with an IQCODE-SF score of 3.2, indicating cognitive decline. At 6 months, this group showed improvement and returned to the cognitive score reported before admission. The delirium patients had the highest scores from admission to the 3- and 6-month follow-ups, indicating more severe cognitive dysfunction (Figure 3).

## 4. Discussion

Our study evaluated the trajectories of early SSD and its impact on clinical outcomes and post-discharge follow-ups in critically ill patients. We found that 24.5% of these patients had early SSD, which was associated with significantly worse clinical outcomes during their ICU stay and at 3-month follow-up. 

Most of the studies on SSD in ICUs have focused on patients who have undergone on-pump cardiac surgery, with reported incidence rates ranging from 34% to 57.1% [10,19,31], which are higher than the prevalence observed in our study. There was one study by Serafim R. et al., which examined early SSD in ICUs, using the CAM-ICU scale in a retrospective analysis, and it reported a prevalence rate of 22.7%, which is very similar to our findings [21]. We observed considerable differences in the methodological approaches used to calculate the prevalence of SSD, which may explain the discrepant results across studies. As an example, in a similar study to ours, Breu et al. considered patients who presented with SSD and delirium, but at different examination time points, in both groups [31]. These differing strategies make it difficult to compare results, as mentioned in previous systematic reviews on SSD [17,32]. 

We used the ICDSC scale; however, neither this scale nor any other has been validated specifically for the clinical identification of SSD in ICUs. Evidence has suggested that the use of quantitative tools such as the ICDSC could aid with SSD assessment, as part of a presentation in a graduation of cognitive dysfunction. With the ICDSC, SSD is identified when the total score falls between 1 and 3 points. This means that, between 0 points (no symptoms) and 4 points (indicative of delirium), there is a range of presentations that are minor in number (as compared to delirium), but could be very relevant as clinical presentations and related to worsening outcomes. The Confusion Assessment Method for the ICU (CAM-ICU) is a dichotomous scale, primarily designed for delirium assessment (present/absent) [33]. In some studies, the CAM-ICU has been used for SSD assessment, where SSD is considered to be present if the CAM-ICU is negative, but the patient exhibits at least one CAM-ICU feature. CAM-ICU is a binary approach to delirium detection, which does not provide a predictive measure of cognitive dysfunction severity, hindering the potential for early interventions. This limitation may be addressed by the new delirium-rating scale, the CAM-ICU-7, which offers a graded scale for assessing the delirium severity.

In our study, we did not assess the use of sedative medications on a daily basis (see Supplementary Materials, Table S1). However, in future research, this is an important area to investigate. Some patients may exhibit features of SSD, such as an altered consciousness, leading to a 1-point score on the ICDSC, which could be a result of residual sedation. Therefore, it is imperative to exclude recent sedative use to ensure that this altered state of consciousness is not an effect of sedation, thus ensuring a correct SSD diagnosis. 

The relevance of an RASS score of −3 in delirium diagnosis has been approached differently in various studies. In some instances, researchers have chosen to apply delirium scales only to patients with an RASS score of ≥−2, as they believe that reliable results cannot be obtained for patients with an RASS score of −3 using any of the validated diagnostic tools [34]. In the context of SSD, there have been no studies examining the impact of sedation on SSD assessment. Furthermore, the validity of the ICDSC has been shown to be considerably better for non-sedated patients, specifically those with an RASS score of 0 or −1 [35].

The patients with SSD were older and had greater critical illness (assessed using the APACHE II and SOFA scores) than the no delirium/SSD patients. In the study by Ouimet et al., the patients with no delirium/SSD were younger, and also had the lowest APACHE II scores at admission when compared to the SSD and delirium patients, respectively [36]. These patients were more likely to be discharged home and less likely to need convalescence and long-term care than those with SSD. Highest admission APACHE II has been associated with SSD in various studies, resembling those described for delirium [14,19,37,38]. In addition, Serafim R. *et* al., studying the impact of SSD occurrence and its trajectory during ICU stays, reported that patients with delirium or coma had a higher baseline severity of illness (expressed by a higher APACHE II score), and an APACHE score of >23 points was one of the main factors associated with the progression from SSD to delirium or coma [21]. Additionally, Vicente Cés Souza-Dantas et al., in 629 critically ill patients under mechanical ventilation, managed to identify an association between the duration of acute brain dysfunction and the baseline levels of C-Reactive Protein (CRP) and SAPS II (sensitivity and specificity of 80%) [39]. 

Early SSD was found to be associated with a prolonged ICU length of stay (LOS) and an increased duration of mechanical ventilation. These outcomes were more similar to the group without delirium/SSD, although these results are limited by our small sample size. The number of days on mechanical ventilation in both the no-delirium/SSD and SSD groups showed a median interquartile range (IQR) between 0 and 1 day. However, a significant percentage of patients in both groups received invasive mechanical ventilation, with 28.3% in the no-delirium/SSD group and 50% in the SSD group. While we did not conduct a specific analysis on the ventilated patients, we can hypothesize some reasons for the short duration of mechanical ventilation in these groups: these patients were younger, including more admissions from the operating room and elective surgeries, and exhibited a better performance status compared to the patients with full delirium.

The trajectory of SSD in ICUs has been analyzed in various studies and documented as follows: (1) SSD patients can revert to a normal cognitive state or (2) deteriorate into delirium or coma. Furthermore, SSD can either (1) be the initial presentation of brain dysfunction or (2) exist along a continuum with delirium (delirium improving into SSD or SSD worsening into delirium). The trajectory involving delirium or coma tends to be associated with poorer outcomes. In our study, we were unable to establish a clear cognitive pathway from SSD to delirium, as only 1 SSD patient (3.8%) progressed to delirium, while 12 patients with delirium (35.3%) progressed to SSD. The rapid resolution of SSD on the 3rd, 4th, and 5th days was more similar to the patterns of patients without delirium/SSD. Serafim R. et al. proposed that the trajectory of SSD should influence outcomes, and their study revealed that SSD often represents a transitional phase that rapidly evolves towards either normalization or deterioration into delirium or coma [21]. SSD patients who deteriorated into delirium or coma experienced longer ICU lengths of stay compared to those who improved or maintained their mental status (8 (5–11) vs. 6 (4–8) days, *p* = 0.025), but this did not lead to an increased mortality [21]. The key factors associated with the progression from SSD to delirium or coma were the use of mechanical ventilation, intravenous benzodiazepine administration, and a baseline APACHE II score of >23 points.

We documented that early SSD was not found to be associated with hospital and ICU mortality, which aligns with the findings of previous studies [21]. However, we acknowledge that assessing ICU-free days may provide a greater sensitivity than mortality in capturing the outcomes of critically ill SSD patients, although this analysis was not performed in our study.

Regarding functional outcomes, most SSD patients maintained their independence in the successive evaluations, with a slight reduction at the 3-month follow-up, without statistical significance. In contrast, the patients with delirium consistently showed higher levels of dependency. This functional presentation of the SSD patients was also more similar to that of the no-delirium/no-SSD group rather than that of the delirium group. 

Concerning cognitive outcomes, at admission, the SSD patients exhibited an IQCODE-SF score of 3.13, which already indicated cognitive decline. This was in initial IQCODE testing, and further testing with a more specific tool is necessary for cognition and dementia assessments. This score was more similar to the patients without delirium/SSD, who had a score of 3.05. During the post-discharge follow-up at 3 and 6 months, it was observed that the SSD patients experienced cognitive impairment that persisted for at least 6 months, with greater impairment reported at 3 months. In a study conducted by Li et al., similar findings were reported in patients undergoing elective coronary artery bypass graft surgery [19]. They observed intermediate cognitive scores in the SSD patients (compared to those with delirium and no symptoms), assessed by the Mini-Mental State Examination, with 1.2 points dropped from admission to hospital discharge and improvement up to 2.6 points from discharge to 2 to 4 weeks later.

During critical illness, alterations in cognition can be recognized and manifest in various patterns. Some patients may experience a worsening of pre-existing cognitive dysfunction, while others may develop new cognitive impairments or not exhibit cognitive dysfunction at all. The recognized risk factors for cognitive dysfunction during critical illness include: advancing age, pre-existing cognitive disorders, severe sepsis, and a longer duration of mechanical ventilation and longer duration of delirium [40]. 

Delirium is the most common manifestation/ marker of acute brain dysfunction during critical illness, and the longer a patient suffers from delirium, the greater the chance of long-term cognitive impairment [29]. Like delirium, SSD should be considered as a manifestation of acute brain dysfunction, but with a less severe presentation.

In our study, the delirium patients presented higher cognitive dysfunction scores, with the highest score seen at 3 months. Pandharipande et al. demonstrated an association between duration of delirium and cognitive dysfunction at 3 and 12 months post-discharge (*p* = 0.001 and *p* = 0.04, respectively) and worse executive function at 3 and 12 months (*p* = 0.004 and *p* = 0.007, respectively) [29]. The fact that delirium and long-term cognitive dysfunction were documented in young patients with a low likelihood of prior cognitive disease suggests that these dysfunctions were acquired during the acute episode in the ICU. They concluded that a longer duration of delirium was associated with worse long-term global cognition and executive function, an association that was independent of the sedative or analgesic medication used, age, preexisting cognitive impairment, the burden of coexisting conditions, and ongoing organ failures during ICU care. This study brings relevance to the importance of delirium in cognition. Subsyndromal Delirium (SSD) may share similarities with delirium and, as documented in our study, may contribute to cognitive impairments post-discharge, but with a less severe presentation than that of delirium.

Comparing studies on cognitive dysfunction is hindered by the lack of consensus on a definition of cognitive dysfunction and the scales used. Another limitation in various studies is the limited information on overall health and pre-hospitalization cognitive statuses for follow-ups and defining the course of progression.

To the best of our knowledge, this is the first study to evaluate the outcomes of early SSD, including functional and cognitive impairments, after hospital discharge during a 3–6-month follow-up period. The decline in cognition and functional capacities is higher at 3-month follow up, but seems reversible, with an improvement or even resolution at 6-month follow-up. Therefore, concerning the higher risk during the first 3 months after discharge, rehabilitation efforts are of particular interest and benefit as soon as the patient is discharged, and should be prepared before patients are discharged. 

The limitations of our study must also be acknowledged. The primary limitation is its small sample size. Due to the scarcity of studies specifically focusing on SSD in mixed ICUs, it was challenging to estimate an adequate sample size, and our sample may have been insufficient for detecting significant differences between the groups. Therefore, larger studies specifically designed to evaluate SSD are necessary to validate our findings.

Secondly, we chose to include patients with early SSD as their initial presentation to minimize the influence of multiple factors associated with prolonged ICU stays. However, it should be noted that some patients may have continued to experience SSD beyond this period or developed SSD after a period of delirium. Furthermore, post-operative delirium is known to occur within the first two days after surgery, but our study only had a 27.4% representation of surgical patients, limiting our ability to address this aspect [41]. A third limitation is related to the frequency of the delirium assessments. Given the fluctuating nature of delirium, a single daily assessment may not be sufficient to capture its presence or absence accurately. Additionally, the assessments were performed by a clinician with the appropriate training, but we did not assess interobserver agreement, which should be considered to be a limitation of the study. Finally, follow-up assessments at 3 and 6 months were conducted with the patients themselves and their proxies. Family involvement can introduce potential biases, as their interpretation of the patient’s limitations may differ from the patient’s self-perception. This limitation is acknowledged in this study.

## 5. Conclusions

The early occurrence of SSD is a prevalent condition, with intermediate outcomes when compared to delirium and patients without SSD or delirium. It is associated with an increased use of resources (regarding the association with an increased duration of mechanical ventilation and ICU length of stay), but not with ICU or hospital mortality. In the long-term follow-up, despite findings of a worsening functionality and cognition at 3 months, most of the patients improved within 6 months. This shows that this condition is relatively “benign” in the medium term, with transitory worsening outcomes at 3-month follow-up. 

These findings emphasize the importance of regular assessments for SSD, the optimization of patient care during ICU stays, and preparation for patient discharging with an early rehabilitation plan. Given the limited studies on interventions aimed at reducing SSD, it is advisable to implement the same non-pharmacological interventions that are well-documented for minimizing the occurrence of delirium.

## Figures and Tables

**Table 1:** Demographic and clinical variables of patients according to the worse mental status in the first 72 h.

	No-Delirium/No-SSD (n = 46)	SSD (n = 26)	Delirium (n = 34)	*p*-Value
Male, n (%)	26 (56.5%)	13 (50%)	21 (61.8%)	0.66
Age, yr. mean (dp)	61 (18)	67 (16)	74 (12)	0.003
**Location before ICU, n (%):**				0.02
Emergency department	27 (58.7%)	10 (38.5%)	15 (44.1%)	
Hospital ward	7 (15.2%)	2 (7.7%)	10 (29.4%)	
Operation room	12 (26.1%)	11 (42.3%)	5 (14.7%)	
Other hospital	0	3 (11.5%)	4 (11.8%)	
**Admission characterization:**				0.09
Medical	25 (54,3%)	15 (57.7%)	26 (76.5%)	
Surgical	12 (26.1%)	9 (34.6%)	8 (23.5%)	
Trauma	8 (17.4%)	2 (7.7%)	0	
Neurological	1 (2.2%)	0	0	
**Admission type, n (%):**				0.17
Urgent	43 (93.5%)	23 (88.5%)	34 (100%)	
Elective	3 (6.5%)	3 (11.5%)	0	
**Admission diagnosis, n (%):**				-
Infection disease/sepsis	15 (32.6%)	9 (34.6%)	15 (44.1%)	
Respiratory failure	1 (2.2%)	3 (11.5%)	4 (11.8%)	
Renal failure	3 (6.5%)	0	0	
Shock	5 (10.9%)	2 (7.7%)	6 (17.6%)	
Metabolic disturbance	3 (6.5%)	0	2 (5.9%)	
Cardiac Arrest	1 (2.2%)	1 (3.8%)	2 (5.9%)	
Trauma	8 (17.4%)	2 (7.7%)	1 (2.9%)	
Urgent Surgery	5 (10.9%)	3 (11.5%)	4 (11.8%)	
Elective Surgery	3 (6.5%)	4 (15.4%)	0	
Other	2 (4.3%)	2 (7.7%)	0	
Charlson Index, median (IQR)	1 (0–3)	2 (0–4)	3 (1–5)	0.17
APACHE II, median (IQR)	15 (10–22)	20 (10–25) (*p* = 0.02) ^+^	26 (20–29) (*p* = 0.02) ^+^	<0.001
SOFA score, median (IQR)	5 (4–7)	6 (5–8)	7 (6–10)	<0.001
E-PRE-DELIRIC score median (IQR)	0.56 (0.33–0.73)	0.58 (0.47–0.68) (*p* < 0.001) ^+^	0.83 (0.66–0.89) (*p* < 0.001) ^+^	<0.001
ECOG Performance status n (%)				0.13
0–1	38 (82.6%)	24 (96.0%)	24 (70.6%)	
2	5 (10.9%)	1 (4.0%)	5 (14.7%)	
3–4	3 (6.5%)	0	5 (14.7%)	

Legend: ^+^—Bonferroni test between SSD and Delirium; SSD—Subsyndromal delirium; ICU—Intensive Care Unit; ICDSC—Intensive Care Delirium Screening Checklist; and ECOG—Eastern Cooperative Oncology Group.

**Table 2:** Patients’ clinical outcomes.

	No-Delirium/No-SSD (n= 46)	SSD (n = 26)	Delirium (n = 34)	*p*-Value
Invasive mechanical ventilation n (%)	13 (28.3%)	13 (50%)	20 (58.8%)	0.02
Days on mechanical ventilation median (IQR)	0 (0–1)	0.5 (0–1)	1.5 (0–7)	0.003
Length of ICU stay (days) median (IQR)	4 (2–6)	4.5 (3–6)	8 (4–10)	0.002
Length of Hospital stay (days) median (IQR)	17 (11–36)	20.5 (9–39)	23 (16–41)	0.22
Hospital mortality	6 (13.0%)	6 (23.1%)	8 (23.5%)	0.41
ICU mortality	2 (4.3%)	0	2 (5.9%)	0.68
Renal Replacement Therapy	5 (10.9%)	5 (19.2%)	10 (29.4%)	0.11
CRP, median (IQR)	9 (6–17)	10 (5–14)	10 (6–14)	0.77

Legend: CRP—C-reactive protein; SSD—Subsyndromal delirium; and ICU—Intensive Care Unit.

**Table 3:** Vital status, re-admission, and location at 3 and 6 months after hospital discharge.

	3 Months				6 Months			
	No	SSD	Delirium		No	SSD	Delirium	
**Alive**	36 (97.3%)	16 (100%)	20 (90.9%)	*p* = 0.43	32 (94.1%)	14 (87.5%)	15 (100%)	*p* = 0.35
**Dead**	1 (2.7%)	0	2 (10%)		2 (5.9%)	2 (12.5%)	0	
**Hospital** **Re-admission**	8 (22.2%)	2 (13.3%)	5 (29.4%)	*p* = 0.63	5 (15.6%)	0	6 (40%)	*p* = 0.02
**Home**	32 (88.9%)	14 (93.3%)	12 (70.6%)	*p* = 0.07	31 (96.9%)	13 (92.9%)	14 (93.3%)	*p* > 0.99
**Nursing home**	1 (2.8%)	1 (6.7%)	1 (5.9%)		1 (3.1%)	1 (7.1%)	1 (6.7%)	
**Long-term care facility**	3 (8.3%)	0	1 (5.9%)		0	0	0	
**Hospital**	0	0	3 (17.6%)		0	0	0	

Legend: SSD—Subsyndromal delirium; No—No delirium/No-Subsyndromal delirium.

**Table 4:** Katz Index of Independence in Activities of Daily Living at admission, 3, and 6 months after discharge.

	Admission			3 Months			6 Months		
	No- n = 46	SSD n = 26	D n = 34	No- n = 36	SSD n = 15	D n = 18	No- n = 32	SSD n = 13	D n = 13
Total dependency	0	0	5 (14.7)	1 (2.8)	3 (20)	3 (16.7)	1 (3.1)	0	2 (15.4)
Severe dependence	1 (2.2)	0	2 (5.9)	4 (11.1)	2 (13.3)	6 (33.3)	3 (9.4)	0	4 (30.8)
Moderate dependence	4 (8.7)	0	4 (11.8)	7 (19.4)	0	3 (16.7)	1 (3.1)	0	0
Mild dependence	5 (10.9)	1 (3.8)	2 (5.9)	2 (5.6)	0	0	0	0	0
Total independence	36 (78.3)	25 (96.2)	21 (61.8)	22 (61.1)	10 (66.7)	6 (33.3)	27 (84.4)	13 (100)	7 (53.8)
*p*-Value	*p* = 0.02			*p* = 0.06			*p* = 0.04		

Legend: SSD—Subsyndromal delirium; No—No delirium/No-Subsyndromal; and D—Delirium.

## Data Availability

The data presented in this study are available on request from the corresponding author. The data are not publicly available due to ethical reasons.

## Supplementary Materials

The following supporting information can be downloaded at: https://www.mdpi.com/article/10.3390/jcm12196363/s1, Figure S1: Vital status at 3 and 6 months after hospital discharge, SSD—Subsyndromal delirium; Table S1: Medication used at least once, during the study timeline.
