# Supplementary material for: Subsyndromal Delirium in Critically Ill Patients—Cognitive and Functional Long-Term Outcomes
Source: J Clin Med. 2023 Oct 4;12(19):6363. doi: 10.3390/jcm12196363 (PMC10573694; doi:10.3390/jcm12196363)
Supplement: Supplementary file 1 [file jcm-12-06363-s001.zip › jcm-2607848-supplementary.pdf]

Supplementary material:

Table S1. Medication in use, used at least once, during the study timeline.

	No-delirium/no-SSD (n=46)	SSD (n=26)	Delirium (n=34)	*p*-Value
Alfentanil	0	1 (3.8%)	1 (2.9%)	0.504
Ketamine	1 (2.2%)	0	0	>0.999
Dexmedetomidine	0	0 (p=0.048)[+]	7 (20.6%) (p=0.048)[+]	<0.001
Diazepam	0	0	0	-
Fentanyl	1 (2.2%)	1 (3.8%)	1 (2.9%)	>0.999
Haloperidol	0	0	4 (11.8%)	0.012
Midazolam	3 (6.5%)	1 (3.8%)	4 (11.8%)	0.526
Morphine	11 (23.9%)	6 (23.1%)	9 (26.5%)	0.947
Propofol	11 (23.9%)	12 (46.2%)	17 (50%)	0.035
Remifentanil	2 (4.3%)	3 (11.5%)	11 (32.4%)	0.002
Corticosteroid therapy	13 (28.3%)	4 (15.4%)	15 (44.1%)	0.052

Legend: [+] Bonferroni test between SSD and Delirium; SSD- Subsyndromal delirium.

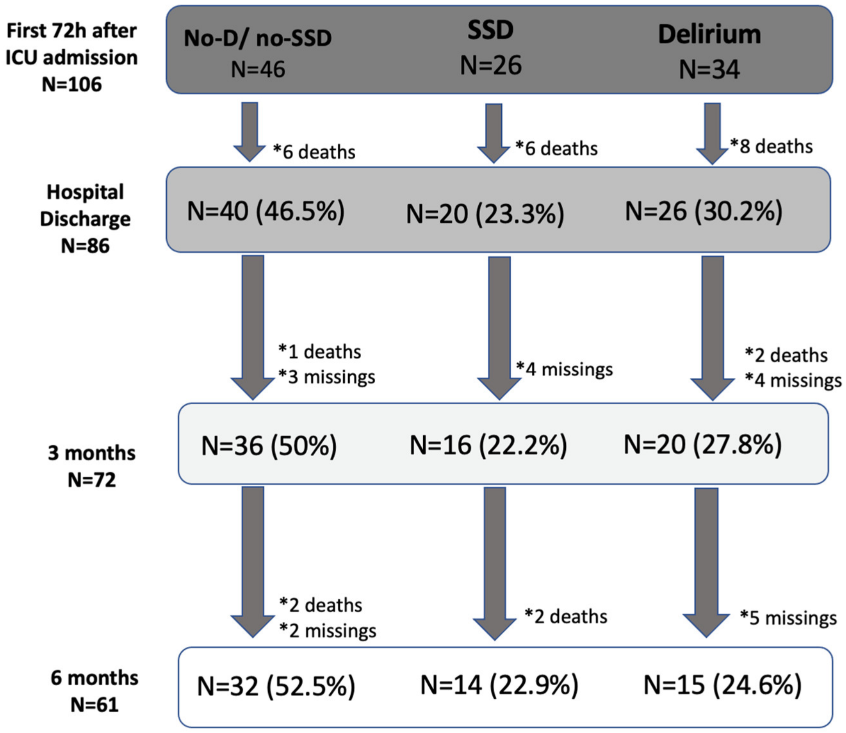

Figure S1. Vital status at 3 and 6 months after hospital discharge; SSD- Subsyndromal delirium.
